# Rethinking the Mechanisms Underlying the McGurk Illusion

**DOI:** 10.3389/fnhum.2021.616049

**Published:** 2021-04-01

**Authors:** Mariel G. Gonzales, Kristina C. Backer, Brenna Mandujano, Antoine J. Shahin

**Affiliations:** ^1^Department of Cognitive and Information Sciences, University of California, Merced, Merced, CA, United States; ^2^Department of Psychology, California State University, Fresno, Fresno, CA, United States

**Keywords:** McGurk illusion, audiovisual fusion, cross-modal phonetic encoding, multisensory integration, phonemic representations

## Abstract

The McGurk illusion occurs when listeners hear an illusory percept (i.e., “da”), resulting from mismatched pairings of audiovisual (AV) speech stimuli (i.e., auditory/ba/paired with visual/ga/). Hearing a third percept—distinct from both the auditory and visual input—has been used as evidence of AV fusion. We examined whether the McGurk illusion is instead driven by visual dominance, whereby the third percept, e.g., “da,” represents a default percept for visemes with an ambiguous place of articulation (POA), like/ga/. Participants watched videos of a talker uttering various consonant vowels (CVs) with (AV) and without (V-only) audios of/ba/. Individuals transcribed the CV they saw (V-only) or heard (AV). In the V-only condition, individuals predominantly saw “da”/“ta” when viewing CVs with indiscernible POAs. Likewise, in the AV condition, upon perceiving an illusion, they predominantly heard “da”/“ta” for CVs with indiscernible POAs. The illusion was stronger in individuals who exhibited weak/ba/auditory encoding (examined using a control auditory-only task). In Experiment2, we attempted to replicate these findings using stimuli recorded from a different talker. The V-only results were not replicated, but again individuals predominately heard “da”/“ta”/“tha” as an illusory percept for various AV combinations, and the illusion was stronger in individuals who exhibited weak/ba/auditory encoding. These results demonstrate that when visual CVs with indiscernible POAs are paired with a weakly encoded auditory/ba/, listeners default to hearing “da”/“ta”/“tha”—thus, tempering the AV fusion account, and favoring a default mechanism triggered when both AV stimuli are ambiguous.

## Introduction

The classic McGurk illusion (McGurk and MacDonald, 1976) is a perceptual phenomenon whereby watching a person utter the consonant vowel (CV) syllables/ga/or/ka/paired with the sounds of/ba/or/pa/may induce illusory auditory perception of a third syllable, “da” or “ta,” respectively. Hearing a third CV has been emphasized as evidence of audiovisual (AV) fusion. Several studies have shown that the McGurk illusion, and by extension AV fusion or integration, occur in a multisensory hub, such as the posterior superior temporal sulcus/gyrus (pSTS/G) or the superior parietal lobule (Calvert et al., 2000; Beauchamp et al., 2004, 2010; Molholm et al., 2006; Senkowski et al., 2006; Erickson et al., 2014). The potency of the McGurk illusion is highly variable among individuals, with some listeners always perceiving it but others rarely experiencing the illusion (Mallick et al., 2015; Brown et al., 2018).

The visual phonemes (visemes) of the classical McGurk illusion (i.e.,/g/and/k/) have an indiscernible place of articulation (POA) and thus have been differentiated from other AV illusions mediated by visemes with discernible POAs like/fa/or/va/(i.e., mouth movements) (Rosenblum and Saldaña, 1992; Abbott and Shahin, 2018; Alsius et al., 2018; Shahin et al., 2018). In the latter case, the illusory auditory percept matches the percept conveyed by the visual modality, referred to as the visual dominance illusion. Shahin et al. (2018) revealed how the visual dominance illusion is manifested in the auditory cortex (AC), by examining the N1-P2 auditory evoked potentials (AEPs) to AV combinations of/ba/and/fa/(both have visually discernible POAs). First, Shahin et al., showed that the N1-P2 AEPs are suppressed for AV pairs of/ba/or/fa/versus auditory-only (A-only) tokens of the same stimuli, consistent with the suppressive effect of visual context on auditory encoding (Besle et al., 2004, 2008; Van Wassenhove et al., 2005; Shatzer et al., 2018). Second, during illusory perception, the N1 to incongruent AV utterances shifted in amplitude, as if the sound of the visually conveyed syllable was presented instead. This shift mirrored the relative N1 amplitudes for/ba/and/fa/in an A-only setting: N1_/ba__/_ is naturally larger (more negative) than N1_/fa/_. Specifically, when individuals were presented with visual/ba/and auditory/fa/and heard “ba,” the N1 increased in amplitude (became more negative). When individuals were presented with visual/fa/and auditory/ba/and heard “fa,” the N1 became smaller. Based on Shahin et al. (2018) and evidence from prior studies (Besle et al., 2004; Van Wassenhove et al., 2005; Pilling, 2009; Smith et al., 2013), we may conclude that the visual cortex modifies phonetic encoding in the AC.

The Shahin et al., study raises the question of whether the same neural mechanisms drive the classic McGurk illusion as well. That is, if visual dominance underlies the McGurk illusion, then in order for individuals to hear illusory “da” or “ta,” their perception of the/g/and/k/visemes presented in a visual-only context must default to “da” and “ta.” There is prior evidence in support for this hypothesis. For example, studies by Tiippana et al. (2004), Tiippana (2014) reported that the individuals, who heard “ete” for audio/epe/and video/eke/, tended to confuse the syllable “eke” with “ete” in a V-only identification task. Specifically, in the V-only task, these individuals saw “eke” as “ete” 45% of the time and as “eke” 42% of the time. Moreover, Saalasti et al. (2011) showed that individuals, who accurately identified V-only/aka/as “aka” (i.e., not confused with “ata”), also had illusory auditory perception that was dominated by “aka” in response to incongruent AV pairings of audio/ata/and visual/aka/. These studies demonstrate that individual differences in V-only perception are reflected in AV illusory perception and align with a visual dominance account of illusory AV speech perception.

To test our hypothesis, we conducted a behavioral experiment (Experiment1) in which individuals (*n* = 19) were presented with a block of silent videos (visual-only or V-only) of a speaker uttering the experimental CVs/da/,/ga/, and/ka/and the control CVs/ba/,/ha/,/la/,/na/,/sa/, and/ya/. The experimental phonemes/g/and/k/have POAs that are visually indiscernible (velar), while the control CVs have mixed POAs: indiscernible (/h/, glottal;/y/, palatal), somewhat discernible (alveolar,/n/,/l/, and/s/), and highly discernible (/b/, bilabial). Participants were also presented with blocks of randomly inter-mixed AV and A-only trials. On the AV trials, individuals watched the same above-mentioned silent videos combined with audios of/ba/. On the A-only trials, individuals listened to audios of/da/, superimposed onto audios of/ba/, forming a/ba–da/A-only combination. There were other A-only combinations as well (see section “Materials and Methods”). Participants performed an open set task whereby they were instructed to transcribe the syllable they saw in the V-only trials and the syllable they heard in the AV and A-only trials. Finally, to evaluate the robustness of the results of Experiment1, we conducted a second experiment with a similar design but using a different set of stimuli from a different talker (Experiment2).

The purpose of the V-only trials was to test whether the classic McGurk illusion occurs because “da”/“ta” is the default visual percept for CVs with an indiscernible POA. Similarly, for the AV trials, we hypothesized that upon pairing audio/ba/with videos of CVs with indiscernible POAs, illusory auditory perception should default to “da”/“ta.” If these two hypotheses are realized, then the results would provide clear evidence that the McGurk illusion is a case of the visual dominance illusion (i.e., where “da”/“ta” is the dominant V-only percept of visemes with an indiscernible POA), rather than being mediated by a fusion process. In the context of the present study, we use the term “McGurk illusion” to refer to the illusion experienced in response to various AV pairings and not just the classic McGurk illusion described above. Finally, the purpose of presenting superimposed pairs of A-only CVs, was to assess the perceptual encoding robustness of one CV (e.g.,/ba/) relative to another (e.g.,/da/) and link this A-only encoding fidelity with McGurk susceptibility. If the McGurk illusion is due to visually mediated modification of phonetic encoding, then individuals who are more susceptible to the illusion should exhibit weaker/b/phonetic encoding (hear “ba” less than “da” in the/ba–da/A-only complex). That is, when the/b/phoneme is weakly encoded in the AC, the visual input can more easily overcome the encoded/b/phoneme (i.e., the auditory input), thereby facilitating the encoding of the visually conveyed phoneme (illusion). Thus, participants, who have more “da” responses for the/ba–da/A-only stimulus, should also experience the McGurk illusion more often, than participants who perceive “ba” more often for the same/ba–da/stimulus. This hypothesis is consistent with Alsius et al.’s (2018) assertion that susceptibility to the McGurk illusion is more robust for weak auditory consonants, particularly because the/b/consonant is confusable with other voice stops.

## Materials and Methods

### Experiment1

#### Participants

Nineteen individuals (>18 years of age, *M* = 21.84 years, SD = 3.59; 11 females; native or fluent English speakers) participated in this study. There were seven native and 12 non-native English speakers of mixed native language backgrounds (Arabic, Spanish, Tagalog, and Vietnamese). All participants reported normal hearing, normal or corrected-to-normal vision, and no language deficits. Participants were recruited via flyers posted on campus and an internal recruiting system of the University of California, Merced. Prior to participation, all participants provided written informed consent. All experimental protocols were approved by the Institutional Review Board (IRB) of the University of California, Merced, and all methods were carried out in accordance with the guidelines and regulations of the IRB of the University of California, Merced and the Declaration of Helsinki. Participants were monetarily compensated for their participation.

#### Stimuli

The stimuli consisted of silent videos (V-only) and corresponding audios of a female talker (mean *f*_0_ = 199 Hz) uttering nine consonant vowel (CV) syllables:/ba/,/da/,/ga/,/ha/,/ka/,/la/,/na/,/sa/, and/ya/. The talker produced these utterances as naturally as possible, without added emphasis or stress. The videos were cropped, such that the talker’s face was visible from the bridge of the nose to the bottom of the neck; this was done to encourage participants to focus on the talker’s mouth instead of other parts of her face (e.g., her eyes). For each CV, we chose three V-only and five A-only exemplars. The experiment consisted of three stimulus conditions: V-only, AV, and A-only. In the V-only condition, the stimuli were silent videos (three exemplars) of the talker uttering the nine CVs resulting in 27 unique V-only trials. In the AV condition, the stimuli were five/ba/audio exemplars combined with the three V-only exemplars of each of the nine CVs, resulting in 135 unique combinations. To create a new AV pairing, the auditory portion of the original video was removed and replaced with an auditory CV of another video, by temporally aligning the acoustic portion of a second video to the time point of the acoustic onset of the original video. In the A-only condition, to create the superimposed A-only pairings of two different CVs, the CVs’ onsets were temporally aligned. The reason for using superimposed CVs in the A-only manipulation was to assess the perceptual robustness of one CV relative to another, to inform of the relative encoding fidelity of these CVs. Specifically, five tokens of/ba/were each combined with five tokens each of/ba/(/ba–ba/),/da/(/ba–da/),/ga/(/ba–ga/), and/la/(/ba–la/), totaling 100 stimuli. There was another A-only stimulus combination, in which five exemplars of/da/and five of/ga/were combined (/da–ga/). In addition to these 125 A-only samples, 10 randomly selected A-only stimuli were included in the experiment to generate a total of 135 stimuli (equal to the number of AV trials). The first subject had 145 trials for the AV and 125 trials for the A-only condition, due to a glitch in the presentation code, which was subsequently corrected. All audio stimuli were normalized in Adobe Audition to the same sound intensity and were presented at ∼65 dBA sound pressure level (measured by a sound level meter positioned 90 cm from the center of the loudspeakers, where the participants would sit).

#### Procedure

Participants sat in an enclosed room about 90 cm from a 27-inch computer monitor with two loudspeakers situated to each side of the monitor. Prior to the start of the experiment, participants were informed that they would be presented with a series of V-only, A-only, and AV stimuli, and were given instructions to transcribe the syllable they see (V-only) or hear (AV and A-only). The instructions offered examples of a broad list of possible syllables that the participants may (e.g., “ta,” “ga,” and “ya”) or may not (e.g., “ra,” “wa,” and “xa”) hear. The instructions specified that the syllable the subject sees or hears could occur once or be repeated many times, and if they hear more than one syllable at the same time, to only transcribe the most dominant one. In the A-only superimposed CVs condition, both syllables are heard to varying degrees, except when both CVs are the same tokens (please refer to the publicly released versions to experience how they sound). Even when the same two CVs are superimposed, one can still hear two instances of the same CV, as these natural speech sounds cannot be exactly aligned in terms of pitch, envelope, formant transitions, etc. Each participant was given a five-stimulus practice session prior to the V-only block and 10-stimulus practice session prior to the combined AV and A-only block. During the practice sessions, a researcher was present in the room to answer questions. Participants typed their response using a keyboard. Stimuli were presented using Presentation v. 20.3 (Neurobehavioral Systems, Berkeley, CA, United States). The experimental session was divided into six 54-trial blocks. The first block consisted of 54 V-only trials (27 stimuli, each presented twice). Crucially, the V-only block was presented first to avoid bias in their V-only percepts due to preceding AV stimuli. For example, if we were to mix the V-only stimuli with the AV stimuli, and if the AV illusion is dominated by “da”/“ta”/“tha,” then participants may be biased to report more “da”/“ta”/“tha” for the V-only stimuli with indiscernible POAs. This would bias the results toward our visual dominance hypothesis, which would be an experimental confound. Blocks two through six consisted of randomly inter-mixed A-only and AV trials with a total of 54 trials in each block. The order of A-only and AV stimulus presentation was randomized to eliminate potential order effects. An optional two-minute break was offered to participants between each block to mitigate boredom and fatigue.

#### Data Analysis

Logfiles of participants’ responses were transferred to Excel spreadsheets, which were then parsed using custom MATLAB code. Responses were categorized according to the first letter transcribed by the participant (i.e., responses “ba,” “bah,” and “bo” were all included in the response category/b/). An exception to this rule was incorporated into the MATLAB code, to distinguish “ta” from “tha,” and “sa” from “sha” responses. However, “c” responses were grouped with “k” due to their phonetic similarity. The output of this parsing code was a table containing information about the condition, stimulus, and percept for each trial.

For the V-only and AV conditions, we tallied each subject’s number of responses corresponding to each of 14 viseme/phoneme percepts: “b,” “p,” “m,” “d,” “t,” “th,” “g,” “h,” “k,” “l,” “n,” “s,” “sh,” and “y.” Other percepts, not included in the 14 above-mentioned percepts, were classified as “other.” Each subject’s perceptual fidelity was calculated as the number of responses for each percept divided by the total number of responses for a specific visual stimulus (CV) type (/ba/,/da/,/ga/,/ha/,/ka/,/la/,/na/,/sa/, and/ya/). Numerical and graphical labels of these two-dimensional stimulus x percept matrices were produced in MATLAB. Responses (percept percentages) corresponding to the five types of A-only stimuli (/ba–ba/,/ba–da/,/ba–ga/,/ba–la/, and/da–ga/) were calculated in a similar way. For the V-only and AV conditions, we also computed the percentage that each percept was reported across all CV types (/da/,/ga/,/ha/,/ka/,/la/,/na/,/sa/, and/ya/; excluding/ba/). This was done to statistically contrast percept strength for stimuli with visually indiscernible POAs.

Statistics included *t*-tests and Pearson’s correlations, as implemented in MATLAB (Mathworks, Natick, MA, United States) Statistics and Machine Learning Toolbox. The *p*-values were Bonferroni-corrected when appropriate to control for multiple comparisons.

### Experiment2

We also conducted a second experiment aimed to replicate Experiment1’s findings, using stimuli uttered by a different female talker (mean *f*_0_ = 184 Hz). These stimuli exhibited slightly added stress in the visual utterance than those of Experiment1 (see examples of the publicly released tokens). Experiment2 used similar procedures and analyses as Experiment1, except for the following differences: (1) Experiment2 included 12 new participants (age *M* = 21.25 years, SD = 3.5; 8 females, native or fluent English speakers). There were seven native English speakers, four native Spanish speakers, and one native Cantonese speaker. Like Experiment1, all participants provided informed written consent, approved by the IRB of the University of California, Merced. All experimental protocols and methods were approved by the IRB and were carried out in accordance with their guidelines. Participants were monetarily compensated for their participation. (2) Visual stimuli for the V-only condition were limited to the following CVs (six tokens each),/ba/,/da/,/ga/,/la/,/sa/, and/sha/; (3) AV stimuli consisted of the V-only (six tokens per CV) stimuli combined with six tokens of audio/ba/stimuli. (4) The A-only trials were limited to the/ba–ba/,/ba–da/, and/ba–ga/pairs (six exemplars of each presented once). (5) Each V-only stimulus was presented twice (total of 72 trials). (6) The AV and A-only stimuli were randomly presented in blocks 2–5 (81 total trials per block). (7) The CVs were uttered with slightly added stress (unlike in Experiment1). To access the original AV stimuli and participants’ behavioral response log files (unprocessed) for Experiment1 and Experiment2, please see the links provided in the Data Accessibility section below.

## Results

This section is organized by stimulus condition/analysis, and the results of both experiments are presented together to highlight the findings that were (or were not) replicated.

### Visual-Only

**FIGURE 1:**
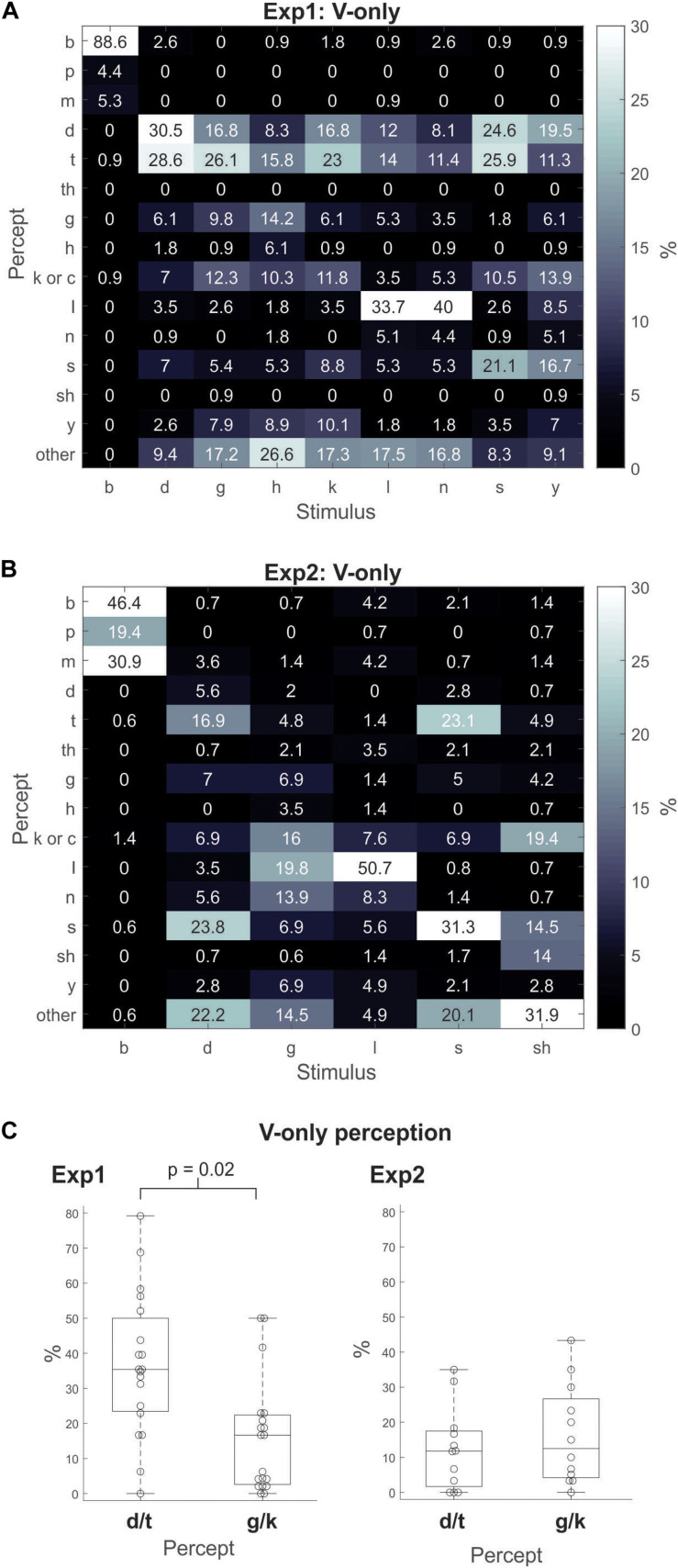
Gray-scale matrix depicting percentages of response percepts to visual-only (V-only) CV utterances for Experiment1 **(A)** and Experiment2 **(B)**. The percentage for each percept was calculated as the percentage of responses of that percept relative to all other responses within a stimulus type (i.e., for each viseme). **(C)** For the visual-only (V-only) CV utterances in Experiment1 (left) and Experiment2 (right), the percentage of responses for the “da”/“ta” or “ga”/“ka” percept relative to all other responses across all visemes, except/ba/. The boxplots indicate the median (the horizontal line inside of the box), the 25 and 75th percentiles (the box’s bottom and top edges, respectively), and the whiskers indicate the range of the individual data points. (Note that there were no outliers).

In Experiment1, we analyzed the data to assess how participants perceived the different V-only stimuli. We calculated the percentage of trials that each percept was experienced within each V-only CV stimulus, e.g., the percentage of “da”/“ta”/“tha” percepts that occurred for the/ga/stimuli,/la/stimuli, etc. Examination of these values (Figure 1A) shows that/ga/and/ka/were identified as “da”/“ta” 43% and 40% of the time, respectively. However,/ga/and/ka/were classified as “ga” or “ka” only about 20% of the time. These results demonstrate that accurate visual identification of/ga/or/ka/(i.e., the visemes used in the classic McGurk illusion) is not robust. Moreover, examination of other CV classifications also yielded substantial “da”/“ta” responses. For example,/ha/was classified 24% as “da”/“ta” and 6% as “ha;”/la/was classified 26% as “da”/“ta” and 34% as “la;”/na/was classified 19% as “da”/“ta,” 4% as “na,” and 40% as “la;”/sa/was classified 51% as “da”/“ta,” and 21% as “sa;”/ya/was classified 31% as “da”/“ta,” and 7% as “ya.”

In Experiment2, the V-only results (Figure 1B) did not show a consistent pattern favoring “da”/“ta” as in Experiment1. For example, identification of “da” and “ta” was not dominant as in Experiment1. In fact,/ga/, was only identified as “da”/“ta” about 7% of the time, and as “ga”/“ka” about 23% of the time – an opposite pattern of the Experiment1 results. Unlike Experiment1 in which the/ba/viseme was identified as “ba” 89% of the time, it was identified as “ba” only 46% of the time in Experiment2. We should note that for both Experiments 1 and 2, the/la/viseme was the stimulus that was most accurately identified, not/da/,/ga/, or/ka/. This is likely due to the fact that the POA of/la/is more discernible than that of/ga/,/ka/, or/da/.

For statistical purposes, we contrasted the instances of “da”/“ta” and “ga”/“ka” in Experiment1 and Experiment2 (separately), by calculating the percent of trials on which each percept was experienced across all stimuli except/ba/. Figure 1C depicts boxplots of the V-only response percentages of “da”/“ta” versus those of “ga”/“ka.” The results show that in Experiment1 (left panel), the “da”/“ta” response significantly dominated the “ga”/“ka” responses: 37% versus 16% [*t*_(__18__)_ = 2.9; *p* = 0.02; Bonferroni corrected]. The same contrast in Experiment2 (right panel) showed no differences between the two percepts: 12% versus 16% [*t*_(__11__)_ = 0.7; *p* = 0.98].

In short, the V-only results of Experiment1 revealed a preference for individuals to default to “da”/“ta” for CVs with indiscernible POAs – even for the/ga/utterance. Results of Experiment2 did not replicate these Experiment1 results. Individuals did not show a tendency to default to “da”/“ta” for V-only CVs with indiscernible POAs. As a matter of fact (although not significant), the CV/ga/was identified as “ga”/“ka” more often than “da”/“ta” overall, in Experiment2.

### Audiovisual

**FIGURE 2:**
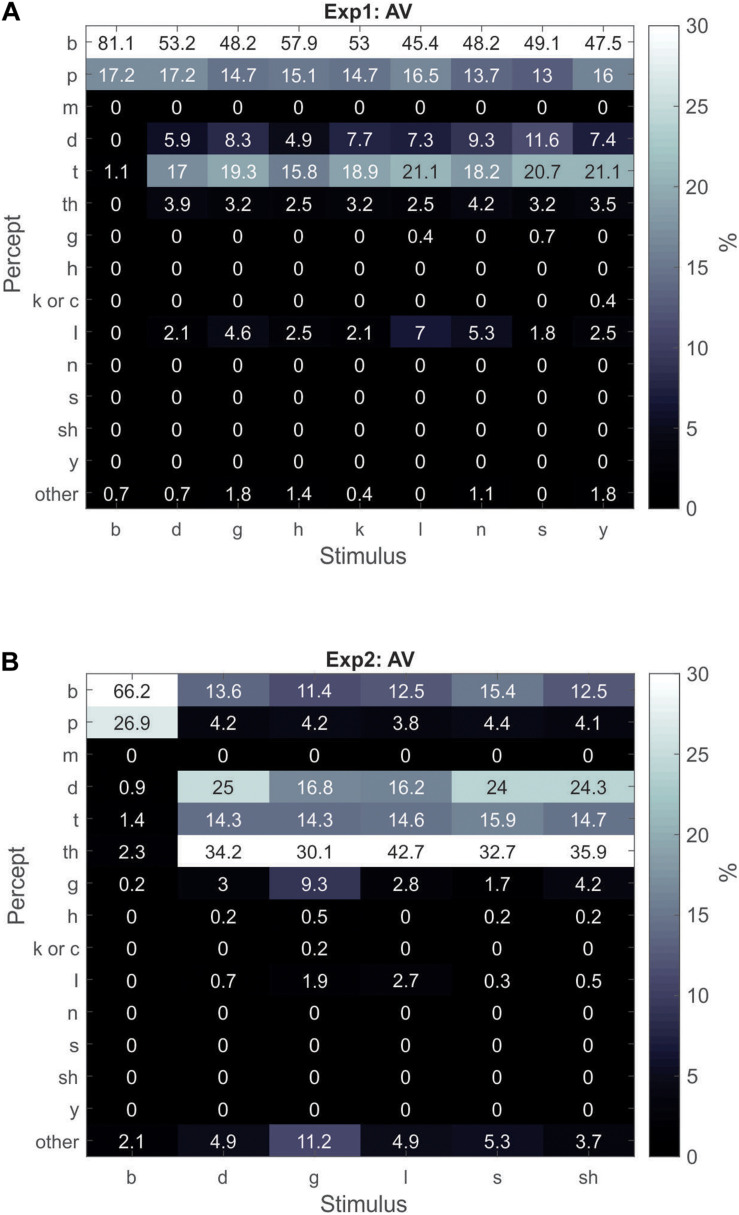
Gray-scale matrix depicting percentages of percepts in response to congruent/ba/AV utterances and incongruent AV utterances of various visual CVs combined with audio/ba/for Experiment1 **(A)** and Experiment2 **(B)**. The percentage for each percept was calculated as the percentage of responses of the percept relative to all other responses within a stimulus type.

Next, the AV responses were analyzed to obtain the percentage of trials each percept was experienced within each CV stimulus type (similar to the V-only analysis shown in Figures 1A,B). In this section and the next section, we also include “tha” as one of the dominant illusory percepts (in addition to “da”/“ta”) and include “pa” as one of the dominant illusory failure percepts (along with “ba”). The “da”/“ta”/“tha” percepts have a very similar manner of articulation and are easily confusable with one another and cumulatively represented the majority of the illusory percepts. Similarly, “ba” and “pa” are highly confusable with one another and represented the majority of the illusion failure percepts. In Experiment1, participants experienced the illusory percept of “da”/“ta”/“tha” across all incongruent combinations about 31% of the time. The Experiment1 results (Figure 2A) revealed that the “da”/“ta”/“tha” percepts dominated participants’ percepts of incongruent AV stimuli when they experienced the illusion. This is in contrast to the V-only condition (Figure 1A), in which participants’ responses were more distributed across the different possible percepts. For example, participants classified/na/as “la” 40% of the time in the V-only condition, but in the AV condition, participants classified the incongruent combination of visual/na/and auditory/ba/31% as “da”/“ta”/“tha” (illusion), 62% as “ba”/“pa” (illusion-failure), and only 5% as “la.” Notably, participants identified V-only/la/as “la” 34% of the time and as “da”/“ta” 26% of the time. Yet, when visual/la/was combined with auditory/ba/in the AV condition, participants’ reported hearing “da”/“ta”/“tha” 31% of the time, but as “la” on only 7% of the trials. This result is surprising given that/l/has a somewhat discernible POA. Similar behavior was observed for other CVs with indiscernible or slightly discernible POAs (e.g.,/sa/and/ga/). Therefore, when auditory information (i.e.,/ba/) is combined with visual CVs of indiscernible or slightly discernible POAs, illusory perception is shifted more in favor of “da”/“ta”/“tha” than when visual speech is presented alone.

For statistical purposes, we calculated the overall percentage of illusory “da”/“ta”/“tha” trials that participants experienced in Experiment1, relative to all other responses of incongruent AV combinations (i.e., not including the AV congruent/ba/stimulus). In contrast to the V-only condition, the AV illusory percepts of “da”/“ta”/“tha” were experienced significantly more often than all other percepts combined. After excluding the “ba”/“pa” illusion-failure responses, “da”/“ta”/“tha” was experienced 31%, while all others at 5% [*t*_(__18__)_ = 3.2; *p* = 0.005].

In Experiment2 (Figure 2B), participants experienced the AV illusion of “da”/“ta”/“tha” across all incongruent combinations about 71% of the time – more often than in Experiment1 [*t*_(__29__)_ = 3.8; *p* = 0.007; *t*-test of independent samples by group]. Furthermore, in Experiment2, the most common of these three illusory percepts was “tha.” Again, like in Experiment1, when visual CVs of indiscernible or slightly discernible POAs were combined with audio/ba/, participants perceived “da”/“ta”/“tha” more often than when visual speech was presented alone (Figure 1B). Replicating the results of Experiment1, the AV illusory percepts of “da”/“ta”/“tha” were experienced significantly more often than all other percepts combined. After excluding the “ba”/“pa” illusion-failure responses, individuals experienced “da”/“ta”/“tha” about 71%, while all others were experienced at 12% [*t*_(__11__)_ = 7.7; *p* = 0.00001].

In summary: (1) With respect to the V-only results, Experiments 1 and 2 diverged. The Experiment1 participants tended to report seeing “da”/“ta” most often, when only visual CVs with ambiguous POAs were presented. However, in Experiment2, “da”/“ta” did not dominate participants’ percepts of visual CVs with ambiguous POAs; instead their perception was more distributed across various percepts. (2) For the AV condition, both Experiments 1 and 2 produced convergent results. During illusory perception of incongruent AV stimuli, participants defaulted to “da”/“ta”/“tha” as the dominant auditory percept, when audio/ba/was incongruently paired with visual CVs with indiscernible or slightly discernible POAs.

### Relationship Between Individuals’/ba/Encoding Fidelity and McGurk Susceptibility

**FIGURE 3:**
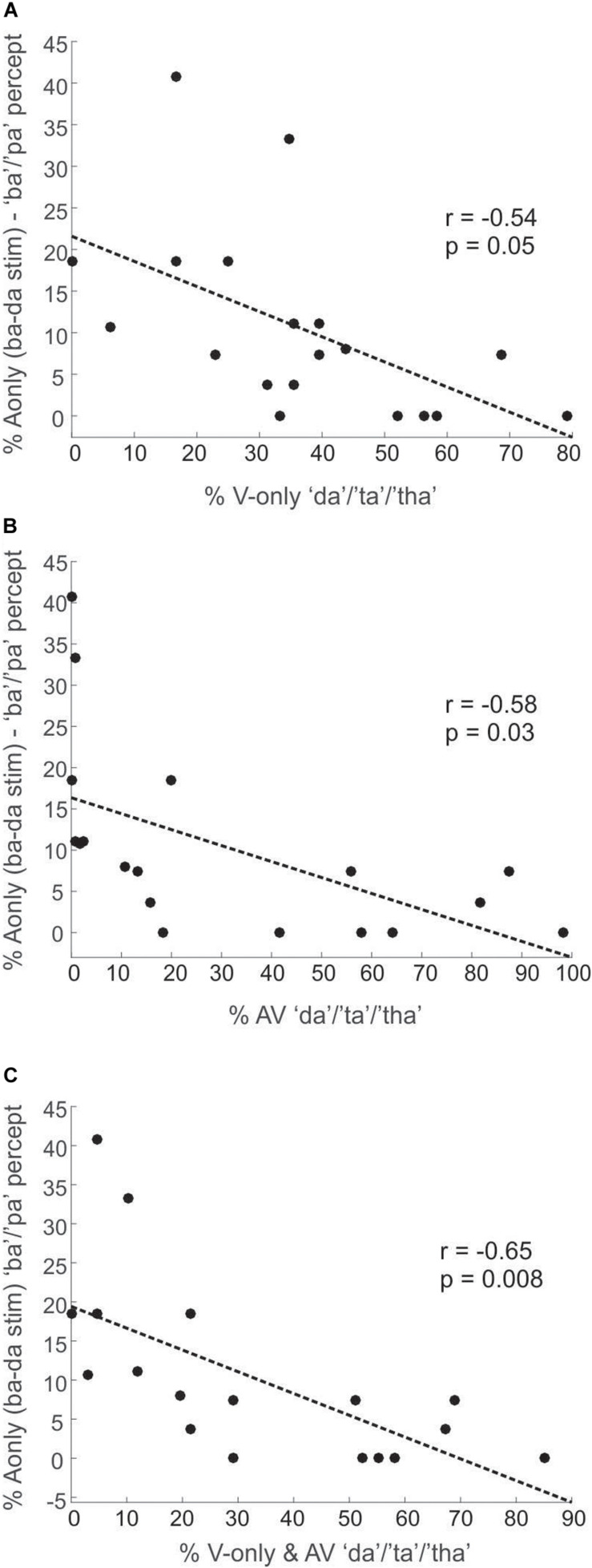
Plots depicting Experiment1 correlations between percentages of the “ba”/“pa” percept of the A-only/ba–da/stimuli (superimposed/ba/and/da/CVs) and the overall response percentages of the “da”/“ta”/“tha” percept (across all stimuli except/ba/) for the V-only condition **(A)**, the AV condition **(B)**, and the mean of the V-only and AV conditions **(C)**.

A second aim of this study was to explore why some people are more susceptible to visually mediated illusory perception (e.g., the McGurk illusion) than others. We hypothesized that individuals, who are more susceptible to the McGurk illusion (across several AV combinations), have weaker phonetic encoding of/ba/than/da/, compared to individuals who rarely experience the McGurk illusion. To test this hypothesis, we conducted across-participants Pearson correlations between the percentage of A-only/ba–da/trials perceived as “ba”/“pa,” and the overall “da”/“ta”/“tha” response percentages to all visemes (except for/ba/), for the V-only (Figure 3A) and AV (Figure 3B) conditions, as well as for the V-only/AV mean (Figure 3C). In this analysis, we excluded responses to the V-only/ba/and AV congruent/ba/stimuli.

We found that the “da”/“ta”/“tha” percept strongly dominated the classification in the A-only/ba–da/condition at 82%, whereas participants perceived “ba”/“pa” on only 11% of these A-only/ba–da/trials. Figure 3 shows the correlation results for Experiment1. We observed significant negative correlations between how often the A-only/ba–da/stimulus was identified as “ba”/“pa” and how often “da”/“ta”/“tha” was visually seen (*r* = −0.54, *p* = 0.05) or audiovisually heard (illusion, *r* = −0.58, *p* = 0.03). We found a similar negative correlation when collapsing the percentage of “da”/“ta”/“tha” responses across both V-only and AV conditions (*r* = −0.65, *p* = 0.008). In other words, participants, who perceived “ba”/“pa” for the A-only/ba–da/stimulus more often, had fewer “da”/“ta”/“tha” responses for the AV and V-only conditions.

**FIGURE 4:**
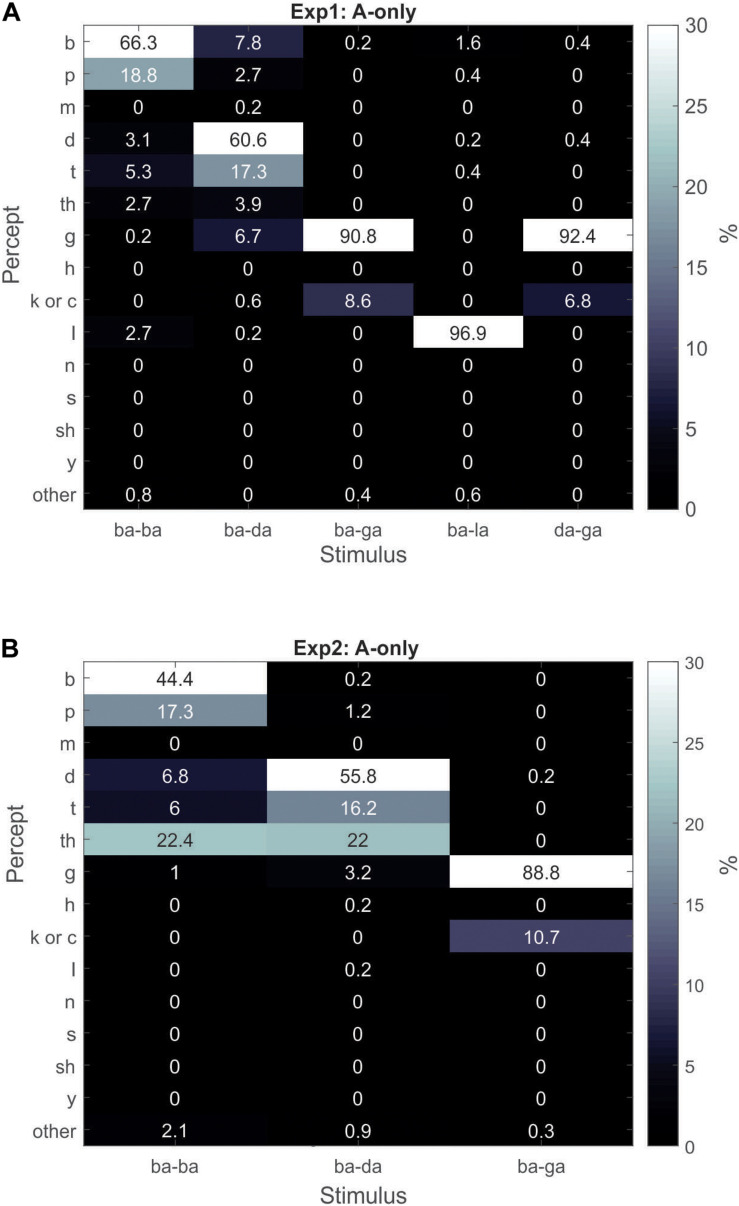
Gray-scale matrix depicting percentages of percepts in response to A-only superimposed stimuli for Experiment1 **(A)** and Experiment2 **(B)**.

In Experiment1, correlating other responses of the A-only combinations (/ba–la/,/ba–ga/, and/da–ga/) with the “da”/“ta”/“tha” response percentages of the V-only and AV conditions did not yield significant results. Moreover, “la,” “ga,” “ba,” and “ga,” response percentages were above 85% for the A-only/ba–la/,/ba–ga/,/ba–ba/, and/da–ga/combinations (Figure 4A), respectively. As a side note, the “ga” percept was overwhelmingly dominant over the “da” percept in the A-only/da–ga/condition (Figure 4A). This is in contrast to the V-only condition in which the/ga/utterance was predominately seen as “da”/“ta” in Experiment1 (but not in Experiment2).

We attempted the same correlations in Experiment2, however, no significant correlations were found, nor would they have made sense given that 9 out of 12 participants had zero “ba”/“pa” responses to the A-only/ba–da/stimuli. The average “ba”/“pa” percent response to the A-only/ba–da/stimuli was 1.4% in Experiment2, compared to 11% in Experiment2 (Figure 4B), compared to 11% in Experiment1. Nonetheless, the rare occurrence of “ba”/“pa” responses to the A-only/ba–da/stimulus, and the high McGurk susceptibility (71%) in Experiment2 (compared to Experiment1), is aligned with the interpretation that strong McGurk susceptibility is associated with weak/ba/auditory encoding.

### Exposure to the McGurk Is Associated With a Perceptual Phonetic Boundary Shift

**FIGURE 5:**
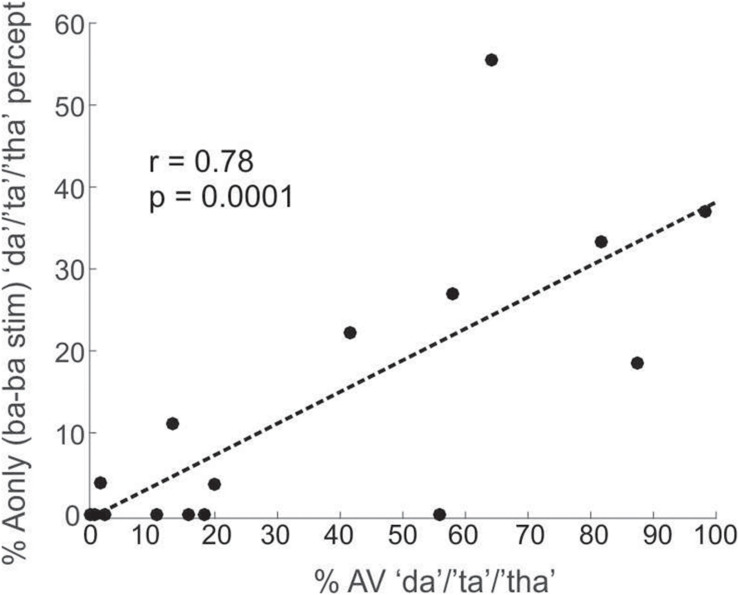
A plot depicting the correlation between percentages of “da”/“ta”/“tha” responses to the A-only/ba–ba/stimuli (superimposed/ba/and/ba/CVs) and the overall response percentages of the “da”/“ta”/“tha” percept during the AV condition. These data are from Experiment1.

The A-only results produced an incidental finding. In the A-only/ba–ba/condition (2 superimposed utterances of/ba/), in which “ba” was the only expected percept, individuals classified the paired CV as “da”/“ta”/“tha” about 11% of the time in Experiment1 and 35% in Experiment2. This result suggests that exposure to the McGurk illusion shifted (recalibrated) listeners’ perceptual phonetic boundary of “ba” toward that of “da”/“ta”/“tha,” at times leading to the auditory percepts of “da”/“ta”/“tha.” Since the McGurk illusion was more robust in Experiment2, it makes sense that this boundary shift was stronger in Experiment2 than in Experiment1. This perceptual shift was correlated with McGurk susceptibility in Experiment1 as shown in Figure 5 (*r* = 0.78, *p* = 0.0001). The same correlation was not significant in Experiment2 (*r* = 0.34, *p* = 0.27), despite strong evidence of perceptual shift (35%, versus 11% in Experiment1). The lack of significance in Experiment2, is likely due to the small number of participants (lack of power). This shift in perceptual boundary due to exposure to the McGurk illusion, is consistent with previous accounts (Bertelson et al., 2003; Kilian-Hütten et al., 2011; Vroomen and Baart, 2012; Lüttke et al., 2016), who showed that AV exposure recalibrates the perceptual boundaries of ambiguous A-only stimuli.

### Native Versus Non-native English Speaker Backgrounds

We finally assessed whether native versus non-native English language backgrounds affected V-only, A-only or McGurk perception, as it has been previously shown that language history could be a factor in AV integration (Hardison, 1996). We used independent samples *t*-tests to determine group (native versus-non-native) differences for the percentages of “da”/“ta”/“tha” in the V-only and AV conditions and for the percentages of the perceptual boundary shifted A-only/ba–da/and/ba–ba/percepts. We collapsed across both experiments to enhance statistical power. There were no significant differences between the native and non-native groups [*t*_(__29__)_ < 1.6 and *p* > 0.12, uncorrected]. Hence, we are doubtful that one’s native language was a factor contributing to the effects reported above. However, all but three of the non-native English participants stated that they learned English at or before age 12. The other three subjects did not provide the time when they began learning English. Thus, lack of non-native language effect in the current data may be attributed to the early age of English acquisition.

## Discussion

Our study revealed four results: (1) V-only perception of a mix of consonant-vowel stimuli is unstable; it has high perceptual variability. (2) When auditory/ba/is paired with various incongruent visemes with indiscernible POAs, the McGurk illusion largely defaults to the percepts “da”/“ta”/“tha.” (3) Individuals with weaker/ba/auditory encoding tend to be more susceptible to the McGurk illusion. (4) Exposure to the McGurk illusion recalibrates the perceptual phonetic boundary, such that the A-only/ba/stimuli that are intermixed with the AV stimuli, are at times perceived as the McGurk percept (“da”/“ta”/“tha”). The results of Experiments 1 and 2 were consistent on findings 2, 3, and 4, but not on finding 1. In Experiment1, during the V-only presentations of/ga/and/ka/, individuals perceived “da”/“ta” more often than “ga”/“ka.” This was not replicated in Experiment2. The lack of compatibility between the V-only results of Experiment1 and 2, is not surprising, due to variability between the talkers.

Both Experiments 1 and 2 confirmed our second hypothesis – that individuals who are more susceptible to the McGurk illusion, display weaker encoding of/ba/as reflected in their perception of the A-only/ba–da/stimulus. This pattern of results could be explained by the following neural mechanism: the weakness in/ba/auditory encoding allows the visual system to overcome the auditory stimulus encoding in favor of the visually conveyed phoneme (Shahin et al., 2018). However, a second factor that may underlie McGurk susceptibility, as implied by the above correlations, is the strength of “da”/“ta”/“tha” visual (not auditory) encoding. If indeed the visual system can overcome phonemes conveyed by the acoustic stimulus in favor of the visually conveyed phonemes (Shahin et al., 2018), then the stronger the “da”/“ta”/“tha” visual encoding, the more likely it can overcome/ba/auditory perception in favor of “da”/“ta”/“tha.”

The relationship between McGurk susceptibility and/ba/encoding fidelity was also observed with the/ba–ba/stimuli. Unlike the/ba–da/A-only stimulus combination, we expected the/ba–ba/stimulus to have no ambiguity whatsoever. Nonetheless, participants still occasionally perceived the A-only/ba–ba/as “da”/“ta”/“tha.” This illusory perception suggests that experiencing the McGurk illusion transfers to A-only settings, in which some individuals dynamically shift their perceptual phonetic boundary from “ba” toward “da”/“ta”/“tha.” This observation is in accordance with prior studies demonstrating that AV exposure can dynamically modulate perception of ambiguous A-only stimuli, through recalibration of perceptual phonetic boundaries (Bertelson et al., 2003; Kilian-Hütten et al., 2011; Vroomen and Baart, 2012; Lüttke et al., 2016). We note, however, that a recent study by Magnotti et al. (2020) did not find correlations between A-only and AV perception. In their study, participants listened to CVs (e.g.,/ba/) embedded in background noise at various signal-to-noise ratios and judged if they heard “ba,” “da,” or “ga.” These participants also made the same perceptual decision on AV (McGurk) trials, during a separate experimental session. Methodological differences—especially whether the A-only and AV stimuli were presented within the same blocks (as in the present study) versus separate sessions (Magnotti et al., 2020)—could explain these divergent results.

Notably, in the present study, both experiments revealed that the McGurk illusion arises because auditory perception tends to default to a few percepts (i.e., “da,” “ta,” or “tha”), when auditory/ba/is incongruently paired with visual stimuli with indiscernible POAs. These findings could not be fully explained by the Fuzzy Logic Model of Perception or FLMP (Massaro, 1987; Massaro and Cohen, 1995; Massaro et al., 1995) and Bayesian-based models (Ma et al., 2009; Andersen, 2015; Magnotti and Beauchamp, 2017). These models emphasize that in AV settings, the weighted probability of the information conveyed by the two modalities drive auditory perception, as described in more detail below.

The FLMP model posits that during AV speech perception, each source of information (auditory or visual) is first evaluated according to the number of alternatives that it can convey. For example, the same viseme or phoneme could inform “*d*” and “*t*” phonemes, but with varying strength. Then, the alternatives of each of the sources (visual and auditory modalities) are evaluated independently from one another, and an overall degree of reliability is assigned to each alternative, according to the level of support they receive from each source. Finally, a perceptual decision is made based on the strength of the overall degree of support for each alternative. The FLMP model’s efficacy was demonstrated in Massaro et al. (1995). They varied the auditory stimuli along the/ba/–/da/formant continuum and the visual stimuli along the/ba/–/da/mouth movement continuum. Participants listened to combinations of auditory and visual stimuli from these continua and reported what they heard. They found that the strength of combined reliability (weights) of the auditory and visual stimuli modulated what participants heard, mostly biased toward “ba,” and “da.” However, the obvious differences between their study and the current one are: (1) the auditory stimulus was held constant in this study, but varied in their study; (2) the present study (also see Lalonde and Werner, 2019) used far more visual speech tokens than in Massaro et al. (1995). It is thus not surprising that the FLMP has been shown to exhibit over-fitting (have high generalization errors) (Andersen, 2015; Andersen and Winther, 2020).

Like the FLMP, the Bayesian integration models (Ma et al., 2009; Magnotti and Beauchamp, 2017) emphasize the significance of stimulus reliability, but also underscore the importance of binding. Magnotti and Beauchamp’s (2017) Causal Inference of Multisensory Speech (CIMS) model incorporates a causal inference decision, which determines whether the auditory and visual input come from the same source (e.g., talker) and thus whether they should be bound. Their model is in line with other reports suggesting a two-stage process in AV integration: binding and fusion (Berthommier, 2004; Nahorna et al., 2012). The CIMS model used the McGurk illusion phenomenon to map an AV phonetic representational space spanning between/ba/and/ga/, with the/da/space situated in-between. The representational spaces along the *y*- (visual) and *x*- (auditory) axis were determined based on behavioral confusability data and prior modeling work. In the CIMS model, following the AV binding stage, the representational probabilities of the auditory and visual information attributed to the same and different sources are then integrated (fusion stage). Consequently, perception could involve hearing either an intermediate percept (McGurk illusion), or a percept that reflects the visual stimulus (visual dominance illusion) or auditory stimulus (auditory dominant, illusion-failure), depending on where the fused representation falls within the representational space. The CIMS model robustly predicted the behavioral outcome for the McGurk inducing AV combination (auditory/ba/and visual/ga/), in which the illusion and illusion-failure are significantly manifested, and the opposite combination (auditory/ga/and visual/ba/), in which the illusion-failure percept overwhelmingly dominates (Magnotti and Beauchamp, 2017).

While the above interpretations of the FLMP and Bayesian frameworks may fit the classic McGurk illusion case, where the representational space map follows a continuous phonetic transformation, it is hard to generalize the predictions of these models to other AV combinations with distant phonetic relationship. For example, in the present study, despite the/l/phoneme having a viseme with strong V-only reliability (high accuracy), individuals still heard the AV combination of visual/la/and audio/ba/as “da”/“ta”/“tha” more often than “la.” One would expect that individuals would attribute visual/la/and auditory/ba/to different sources and thus binding would be weak, yet the default perception to “da”/“ta”/“tha” was still dominant. Furthermore, the phonemes/d/,/t/, or/th/are not intermediate to/l/and/b/in terms of POA (/d/,/t/, and/l/∼ alveolar,/b/bilabial) or formant transition, so their individual probabilities would not fall within an intermediate representational space as with the classic McGurk illusion. This was the case for other CVs with a highly variable mix of POAs and formant transitions and fricatives. Thus, in order for each incongruent AV combination to produce the “da”/“ta”/“tha” auditory percept and satisfy the claims of the Bayesian and FLMP models and the “fusion” account, the weights of the auditory and visual percepts of the incongruent pairs must either (1) always sum to the same value regardless of their individual weights, or (2) if the auditory weight is always constant (e.g., “ba”), as in the current experiment, then the visual percept weight must default to the same value regardless of the visual stimulus. Our results across both experiments do not provide strong evidence that visual dominance (i.e., perceiving a viseme with an indiscernible POA as “da”/“ta”) drives the McGurk illusion, since this effect was not replicated in Experiment2. We are left with alternative explanation, that under AV conditions with incongruent AV stimulus pairs (e.g., visual/ga/and auditory/ba/), the brain defaults to hearing (guesses) “da”/“ta”/“tha” when attempting to associate a weakly encoded auditory/ba/and a weakly encoded visual utterance (i.e., with an indiscernible POA). If the acoustic stimulus is robustly encoded, then no guessing is necessary, i.e., the illusion fails.

In conclusion, findings from both experiments showed that during perception of incongruent AV speech stimuli, individuals default to specific percepts (e.g., “da”/“ta”/“tha”), despite a mix of AV incongruent combinations with differing visual weights (reliability). These findings suggest that the mechanisms that underlie the McGurk illusion are driven by the perceptual ambiguity of both AV stimuli, which may lead to a best guess default percept (i.e., “da”/“ta”/“tha”).

## Data Availability Statement

The datasets presented in this study can be found in online repositories. The names of the repository/repositories and accession number(s) can be found below: https://figshare.com/articles/Experiment_1_Data/11868150. https://fig share.com/articles/Experiment_2_Data/11868357.

## Ethics Statement

The studies involving human participants were reviewed and approved by The Institutional Review Board, University of California. The patients/participants provided their written informed consent to participate in this study.

## Author Contributions

MG, KB, BM, and AJS designed the experiment and analyzed the data and wrote the manuscript. MG and BM performed the experiment. All authors contributed to the article and approved the submitted version.

## Conflict of Interest

The authors declare that the research was conducted in the absence of any commercial or financial relationships that could be construed as a potential conflict of interest.
